# Reaching late adopters: factors influencing COVID-19 vaccination of Marshallese and Hispanic adults

**DOI:** 10.1186/s12889-023-15468-3

**Published:** 2023-04-03

**Authors:** Jennifer L. Vincenzo, Marissa J. Spear, Ramey Moore, Rachel S. Purvis, Susan K. Patton, Jennifer Callaghan-Koru, Pearl A. McElfish, Geoffrey M. Curran

**Affiliations:** 1 College of Health Professions, University of Arkansas for Medical Sciences Northwest, Fayetteville, AR 72703 USA; 2 Office of Community Health and Research, University of Arkansas for Medical Sciences Northwest, Springdale, AR 72762 USA; 3 College of Medicine, University of Arkansas for Medical Sciences Northwest, Springdale, AR 72762 USA; 4 College of Education and Health Professions, University of Arkansas, Fayetteville, AR 72701 USA; 5 College of Pharmacy, University of Arkansas for Medical Sciences, Little Rock, AR 72205 USA; 6 Center for Mental Healthcare and Outcomes Research, Central Arkansas Veterans Healthcare System, North Little Rock, AR 72114 USA

**Keywords:** Socio-ecological model, Faith-based, Community engagement, Vaccine uptake

## Abstract

**Background:**

Marshallese and Hispanic communities in the United States have been disproportionately affected by COVID-19. Identifying strategies to reach late vaccine adopters is critical for ongoing and future vaccination efforts. We utilized a community-engaged approach that leveraged an existing community-based participatory research collaborative of an academic healthcare organization and Marshallese and Hispanic faith-based organizations (FBO) to host vaccination events.

**Methods:**

Bilingual Marshallese and Hispanic study staff conducted informal interviews with 55 participants during the 15-minute post-vaccination observation period and formal semi-structured interviews with Marshallese (n = 5) and Hispanic (n = 4) adults post-event to assess the implementation of community vaccine events at FBOs, with a focus on factors associated with the decision to attend and be vaccinated. Formal interview transcripts were analyzed using thematic template coding categorized with the socio-ecological model (SEM). Informal interview notes were coded via rapid content analysis and used for data triangulation.

**Results:**

Participants discussed similar factors influencing attitudes and behaviors toward receiving the COVID-19 vaccine. Themes included: (1) intrapersonal – myths and misconceptions, (2) interpersonal – protecting family and family decision-making, (3) community – trust of community location of events and influence of FBO members and leaders, (4) institutional – trust in a healthcare organization and bilingual staff, and (5) policy. Participants noted the advantages of vaccination delivery at FBOs, contributing to their decision to attend and get vaccinated.

**Conclusions:**

The following strategies may improve vaccine-related attitudes and behaviors of Marshallese and Hispanic communities not only for the COVID-19 vaccine but also for other preventive vaccinations: 1) interpersonal-level – develop culturally-focused vaccine campaigns targeting the family units, 2) community-level – host vaccination events at convenient and/or trusted locations, such as FBOs, and engage community and/or FBO formal or lay leaders as vaccine ambassadors or champions, and 3) institutional-level – foster trust and a long-term relationship with the healthcare organization and provide bilingual staff at vaccination events. Future research would be beneficial to investigate the effects of replicating these strategies to support vaccine uptake among Marshallese and Hispanic communities.

**Supplementary Information:**

The online version contains supplementary material available at 10.1186/s12889-023-15468-3.

## Introduction

Minority racial and ethnic communities have experienced disparities in COVID-19 infections, hospitalizations, deaths, and vaccination rates [1], [2]. Northwest Arkansas is home to one of the largest populations of Marshall Islanders (Marshallese) outside of the Republic of the Marshall Islands (~ 16,000), and a growing Hispanic community constitutes over 15% of the population in several counties [3], [4]. Inadequate insurance, low-wage employment in essential industries, and housing insecurity in both communities increased their exposure to adverse outcomes from COVID-19 [5]. COVID-19-related disparities among the Marshallese Pacific Islander and Hispanic communities were so significant that the National Institutes of Health and the Centers for Disease Control and Prevention (CDC) conducted an on-site investigation in 2020. The Hispanic and Marshallese communities accounted for 57% of COVID-19-associated deaths, yet Hispanic and Marshallese only represented 17% and 2.4% of the population in the region. The CDC attributed these disparities to confusion regarding COVID-19 prevention, testing, management, language barriers, and decreased awareness or inability to access services [3].

Vaccination is a critical public health strategy for mitigating the effects of COVID-19, but lower vaccination rates in some communities contributed to disparities in COVID-19-related outcomes. In many states, there is a 30–60% difference in vaccination rates between White and Hispanic individuals [6]. Higher rates of vaccine hesitancy among minority communities contribute to lower vaccination uptake, [7] and health insurance coverage, workplace policies, and internet connectivity also contribute to disparities [6], [8]. Among Marshallese, a small survey (n = 120) found that 26.7% did not know or were unsure if they would get vaccinated, and 14.7% reported they were unlikely to do so [5].

Community-based participatory research (CBPR) and community engagement are essential tools to reduce health disparities [9]. To address COVID-19-related disparities in Northwest Arkansas, the University of Arkansas for Medical Sciences (UAMS) leveraged an existing CBPR partnership with the Hispanic and Marshallese communities [9], [10]. CBPR integrates communities into the research process, including research focus, planning, execution, and dissemination, and honors cultural beliefs, social traditions, and community norms [9]. Before the availability of vaccinations, UAMS and CBPR partners developed a community-engaged response to COVID-19, including disseminating culturally appropriate health information, supporting a trilingual contact tracing and case management center, and hosting testing clinics in Marshallese and Hispanic community settings [10]. As vaccines became more widely available in early 2021, efforts shifted to increasing vaccination rates among Hispanic and Marshallese adults by addressing vaccine hesitancy and barriers to access.

According to the World Health Organization (WHO) framework, behavioral and social drivers of vaccine uptake include cognitive and emotional responses, social norms, motivation, and practical issues [11]. A recent survey utilizing the WHO framework in rural India found that vaccine beliefs are impacted by government-elicited vaccine communication strategies, perceived vaccine-related side effects, and trust in the healthcare sector [12]. Similarly, a study conducted in seven Arab countries found that vaccine uptake was related to concerns about side effects, trust in the healthcare system, and the desire to protect others [13]. The socio-ecological model (SEM) posits that individual health behaviors, such as vaccine adoption, are influenced by intrapersonal (e.g., knowledge and attitudes), interpersonal (e.g., relationships and social networks), institutional (e.g., organizational characteristics), community (e.g., social/cultural context), and policy factors (e.g., local, state, or national policies/laws) [14]. Two recent scoping reviews found that all SEM factors played a role in individuals’ decisions to get the COVID-19 vaccine [15], [16]. At the intrapersonal level, knowledge and concerns about the safety of the vaccine affect uptake, while at the interpersonal level, the influence of family and friends affects COVID-19 vaccine acceptance. At the institutional level, media influences and trust in the government, science, and healthcare serve as barriers and facilitators to vaccine uptake. At the community level, community levels of vaccination and COVID-19 infection rates influence vaccination acceptance. Finally, at the policy level, vaccination location, cost of the vaccine, and political influences impact COVID-19 vaccination acceptance. It is essential to understand the broader social, cultural, and political context involved in attitudes and behaviors toward COVID-19 vaccination among different racial/ethnic minority groups to develop targeted strategies to improve vaccine uptake [1]. Yet there remains a gap in the literature regarding factors affecting COVID-19 vaccine uptake among late adopting racial/ethnic minority groups in the context of community-engaged vaccination events. This qualitative study used the SEM framework to identify factors that influenced vaccination attitudes and behaviors of late-adopting Marshallese and Hispanic adults in the context of community-engaged health vaccination events in faith-based organizations (FBO). Our findings will inform the development of culturally relevant strategies to promote vaccine and booster uptake and increase the efficacy of community-engaged health promotion strategies.

## Methods

### Setting and vaccination events

This study occurred in the summer of 2021 during UAMS’ COVID-19 vaccination outreach program with Marshallese and Hispanic communities in Northwest Arkansas, which is described elsewhere [4]. UAMS’ vaccination outreach efforts leveraged an existing CBPR collaboration with the Marshallese and Hispanic communities of Northwest Arkansas [9], [10], [17]. The collaboration facilitated community-engaged strategies to address barriers to access and increase uptake of COVID-19 mitigation services among the Marshallese and Hispanic communities at the beginning of and throughout the pandemic, as described elsewhere [10], [4].

When COVID-19 vaccines became available, UAMS and community partners conducted a rapid needs assessment to understand vaccination barriers. Informational materials, such as fliers and social media messages, were then developed to address specific concerns and barriers. Influential messengers and community partners were trained to deliver information within the Marshallese and Hispanic communities to increase awareness and address vaccination hesitancy. Using a community-engaged approach, a vaccination outreach program was developed, and 39 community-based vaccination events were conducted between 4 May 2021 and 29 October 2021. Most events were held at FBOs with large Marshallese and/or Hispanic congregations and at days/times to facilitate attendance and reduce barriers. Outreach and advertising were conducted by UAMS staff, FBOs, and community partners and included iterative improvements to outreach and advertising efforts as part of continuous quality improvement efforts. Community health workers affiliated with community partners and/or UAMS facilitated access by scheduling appointments and providing resources for transportation for these events.

Vaccination events were staffed by volunteers, clinical staff, community health workers, or other individuals from partnered organizations. All vaccinations were administered by trained clinical staff. Each event included bilingual (English/Marshallese or English/Spanish) volunteers responsible for obtaining patient consent, answering questions, facilitating paperwork, and translating for clinicians and other volunteers. COVID-19 vaccines were provided by the United States (US) Government free of charge to the university and another clinical partner. More than 2,700 doses of the COVID-19 vaccine were administered to attendees across vaccination events.

### Approach

This study follows a pragmatic qualitative case study design [18], [19] to understand influences on vaccination decisions of participants in UAMS’ community-engaged vaccination outreach events at FBOs in Northwest Arkansas. All study materials and procedures were approved by the UAMS Institutional Review Board (IRB #262917), and all participants provided informed consent in their preferred language.

### Participant recruitment

Marshallese and Hispanic adults were recruited to participate at a sample of community engaged FBO vaccination events between July and September of 2021. Recruitment took place at events held in Marshallese (n = 4) and Hispanic FBOs (n = 2), and one event was held in an FBO-affiliated location that provided social, health, and material services (e.g., a food bank, oral healthcare, and home/school supplies). Bilingual Marshallese and Hispanic study staff conducted informal interviews with 55 participants approached during the 15-minute post-vaccination wait time. Study staff also inquired about participant interest in partaking in a longer, in-depth semi-structured interview later. Participants who expressed interest were contacted by email or phone in their language of preference (Marshallese, Spanish, English).

### Data collection

In the initial phase of our study, we conducted brief ethnographic fieldwork involving participant observation and informal interviews with staff, volunteers, and participants [18] at four vaccination events (two Marshallese and two Hispanic-focused). This multi-step qualitative data collection process involved observations and the informal interviews serving as the first step and a more formal semi-structured interview serving as the second step of data collection. This fieldwork and informal interviews with 55 participants allowed us to better understand the implementation context and gain a large breadth of insights into participant motivations for attending the events and aided in preparing semi-structured interview guides for in-depth interviews. We aimed to conduct in-depth semi-structured interviews with ~ 5 Marshallese and ~ 5 Hispanic adults to attain data saturation in phenomenological research [18], [20].

The informal interviews with event participants were brief (3–5 min average duration) and included the following questions: (1) reasons for attending, (2) COVID-19 vaccine decision-making, (3) barriers/facilitators to attendance, (4) recommendations for event improvement, and (5) general thoughts about COVID-19 vaccines (see Additional File 1). Bilingual Marshallese and Hispanic study staff with interview experience conducted informal interviews at vaccination events between July and September of 2021. Interviewers documented informal interviews in one memo per event and debriefed and reviewed memos with study staff after the first two events.

Informal interview participants who expressed interest in participating in in-depth interviews were contacted within 4 weeks to discuss the interview process, and those who agreed were scheduled. Interviews were conducted in the language of the participant’s choice (English, Spanish, or Marshallese) and in person, by phone, or by a secured video-conferencing platform according to participants’ preferences.

In-depth interviews followed a semi-structured interview guide. The guide was developed based on data gathered from the 55 informal interviews with input from the authors (GMC, PAM) and bilingual study staff. Study staff were trained and debriefed on interviews and encouraged to provide recommendations for revisions to the guide. The semi-structured interview guide explored participants’ thoughts on the COVID-19 vaccine, vaccine decision-making, reasons for choosing to attend and get vaccinated at the community location, and recommendations for event improvement (see Additional File 2). Sociodemographic questions from the Behavioral Risk Factor Surveillance System survey were asked to capture participants’ age, educational attainment, employment type, and salary [21], [22]. After study staff completed 1–2 interviews, the team debriefed and made slight revisions to the guides to use with the remainder of the interviews. All interviews were included in the analysis. Interviews lasted between 11 and 41 min. Participants received a $40 gift card as remuneration for their participation. All in-depth interviews were recorded, transcribed, and de-identified before analysis. Marshallese and Spanish transcripts were translated into English by certified translators. Bilingual study staff verified the accuracy of translations before analysis.

### Qualitative data analysis

Rapid thematic analysis of informal interview summaries and in-depth interview transcripts followed a modified Framework approach [19], [23]. The primary analysis team was comprised of two qualitative researchers (JLV and MJS). The team read all summaries and transcripts and created a coding framework and template combining *a priori* codes from the SEM with emergent secondary codes identified during analysis (see Table 1). Formal interview transcripts were independently coded using the template by both researchers, who then met regularly to consolidate the templates (i.e., create one final coded template per participant) and resolve discrepancies in interpretation. Summaries of coded data were transferred to charts with a column for each theme and a row for each participant to facilitate identification of patterns and outliers. Illustrative quotes were identified for each theme. Informal interview memos were also coded in the template and used for data triangulation and to confirm data saturation of the in-depth interviews. The coding framework and template, themes, and findings were critically reviewed by the authors at weekly meetings.

**Table 1:** Operational Definitions of the Socio-Ecological Model

Construct	Operational Definition
Intrapersonal	Individual knowledge, attitudes, and perceptions about the COVID-19 vaccination
Interpersonal	Related to relationships involved in attitudes and behaviors towards the COVID-19 vaccine
Institutional	Related to the impact of the healthcare organization and staff on vaccination attitudes and behaviors
Community	Related to the location of the events and the influence of the community members and community organization on attitudes and behaviors towards the COVID-19 vaccine
Policy	Related to local, state, and national policies and laws about the COVID-19 vaccine

## Results

All informal interviews were with Hispanic and Marshallese participants; however, demographic information of informal interview participants was not collected. Formal interviews were conducted with Marshallese (n = 5) and Hispanic (n = 4) participants, whose self-reported demographic information is provided in Table 2.

**Table 2:** Participant Demographics

Age	Gender	Race/Ethnicity	Education	Income	Job Sector
57	Female	NHPI	Some high school	< $15,000	Cleaning or maintenance
32	Male	NHPI	High school graduate	$25,000 - $49,000	Food processing plant
22	Female	NHPI	High school graduate	Prefer not to answer	Student
35	Male	NHPI	Some high school	$20,000 - $24,999	Food processing plant
54	Female	NHPI	Prefer not to answer	Prefer not to answer	Prefer not to answer
59	Female	Hispanic	Elementary school	$25,000 - $19,999	Don’t know/Not sure
54	Female	Hispanic	Elementary school	Prefer not to answer	Does not work
30	Female	Hispanic	High school graduate	$25,000 - $34,999	Plant and machine operator
34	Female	Hispanic	High school graduate	Prefer not to answer	Don’t know/Not sure

*Note.* NHPI = Includes persons having origin in any of the original peoples of Hawaii, Guam, Samoa, or other Pacific Islands. All participants who reported NHPI were Marshallese.

Both informal and formal interview participants shared their attitudes about the COVID-19 vaccine and factors that influenced their decision to get vaccinated. All participants noted numerous advantages of vaccination delivery through the FBO community-engaged events that contributed to their decision to attend and receive the vaccine. Interpersonal, community, institutional, and policy factors had a positive influence on attendance at events and the decision to get vaccinated. Intrapersonal factors seemed to be associated with more negative attitudes toward vaccination. Factors influencing vaccination-related attitudes and behaviors are elaborated below for each category of the SEM and were similar for Marshallese and His panic participants. Refer to Table 3 for themes, subthemes, and exemplary quotes.

**Table 3:** Summary of Vaccination Influences by Level of the Socio-Ecological Model: Subthemes and Exemplar Quotes

Socio-Ecological Model Level	Subtheme	Exemplary Quote
Intrapersonal	*Myths and misconceptions*	“People who want to twist the information, to say things that aren’t true. They say they’re going to insert a chip, it’s the devil, things like that they invent, right?” (34 y/o Hispanic female)
Interpersonal	*Protecting family and family decision-making*	“Because of this disease, my husband has lost many relatives to COVID. We made the decision of getting vaccinated, because there are many people infected and we need to get a little protected.” (54 y/o Hispanic female)
Community	*Trust of community location of events*	“I would say it’s better [at community location] so we don’t feel embarrassed or get afraid to go somewhere else.” (54 y/o Hispanic female)
	*Influence of faith-based organization members and leaders*	“I heard from our leaders that we must take the vaccine to prevent us [from getting COVID19].” (32 y/o Marshallese male)
Institutional	*Trust in healthcare organization and bilingual staff*	“That was one of the most important things for me. Trust and above all, the same language, so that I can understand. And they can understand me, too.” (28 y/o Hispanic female)
Policy	N/A	“It just seems that it’s going to be mandated eventually along with other vaccines, so I like, might as well.” (30 y/o Hispanic female)

### Intrapersonal

#### Subtheme: Myths and misconceptions

The predominant intrapersonal factor described by formal interview participants was concern about potential side effects driven by misconceptions or misinformation that they heard from other people. One participant stated, “Some were telling me that if I take the COVID-19 vaccine, months or years from now I may develop cancer, kidney problems, these are the things that I have been told that will affect my health” (22 y/o Marshallese female). Other participants mentioned concerns about side effects related to fertility, such as, “My nieces brought it up as well, which I think [they] watched a TikTok saying that [the vaccine] affects you with having babies in the future” (30 y/o Hispanic female). Two participants mentioned rumors about the government inserting a chip through the vaccine, which caused concern about the vaccine. Informal interview participants expressed similar concerns, particularly those around infertility. Less frequently mentioned were concerns about the documented side effects of COVID-19 vaccines (e.g., soreness at the vaccination site and mild fever). The impact of these concerns on attending a vaccination event was not clear from the interviews.

### Interpersonal

#### Subtheme: Protecting family and family decision-making

Interpersonal factors, especially wanting to protect family and family decision-making, were a prominent theme regarding an individual’s decision to get vaccinated. One participant stated, “I want to protect my family so they will not get infected with the Coronavirus and the other new virus that they just found [delta variant]” (22 y/o Marshallese female). Another participant voiced a similar motivation: “I think we have to get conscious about getting vaccinated, for the sake of your neighbor, our family, our children” (54 y/o Hispanic female). Some participants mentioned that they previously lost family members due to COVID-19, which influenced their own or their family’s decision to get vaccinated. For example, a participant said, “That’s one of the reasons why I decided to get [the vaccine], because last year I lost my older brother due to the virus… that’s why almost all of my brothers, my parents are already vaccinated” (34 y/o Hispanic female). Another participant shared that her immediate family members had gotten vaccinated due to her brother’s death from COVID-19, except for her husband who is worried that “it’s a chip they want to put in” (28 y/o Hispanic female).

Participants commonly reported deciding to get vaccinated as a family or that a family member served as an example to get vaccinated. One participant explained, “When they mention at the church…we…signup for the family and grandkids, so we can go take the COVID-19 vaccine” (57 y/o Marshallese female). Another expressed they took their family to get vaccinated, sharing, “My family didn’t want to take the COVID-19 vaccines…but I didn’t want them to get infected” (22 y/o Marshallese female). Others discussed encouragement they received from family members to get vaccinated, such as from a daughter who is a nurse, from a mother, or from an older sister. One participant shared their family’s influence on their decision to get vaccinated:

To be honest, I was very skeptical. I am due for my second dose today. It just seems too new. It was just a little too fast for me. I just postponed it for a year, basically, until, obviously, everything right now… I just felt like I needed to do my part and as well protect myself. It was more of a family thing. First, my parents got it, and then it was one sister after another. We’re like, “Well, we’re all just gonna get it [the vaccine].” We were all just very skeptical about it, but I haven’t got sick, so. (30 y/o Hispanic female)

Only one participant described choosing to be vaccinated despite family opposition:

I decided to take it. My daughters didn’t want me to take it, but I decided to take it with the information I got … I was considering it, The pros and the cons. If I should go or not because my daughters didn’t want me to because they are afraid. They’re not vaccinated yet. I decided to go. I didn’t think too much because the information I have, good or bad, it’s not that much. I just decided to go and avoid getting infected. (59 y/o Hispanic female)

Informal interview participants also reported interpersonal factors as the predominant factor in vaccination decisions. Subthemes were consistent with those of formal interview participants, including protecting family, concerns regarding the loss of family members due to COVID-19, and family decision-making.

### Community

#### Subtheme: Trust of community location of events

Community factors were facilitators for Marshallese and Hispanic participants to attend vaccine events, which included the trust of the community location of the events and the influence of FBO members and leaders. One participant stated, “It’s better in the community and you just feel more sheltered than in another place. You feel more confident in this place” (34 y/o Hispanic female). The same participant described attending the FBO where the priest mentioned the vaccine event and thought, “This is my chance. I didn’t need to make an appointment just to arrive; if anything happened, I knew Spanish was spoken there. So, I thought, I’ll go there and won’t waste this opportunity” (34 y/o Hispanic female). Multiple participants named a community location when asked about where they will receive booster vaccinations in the future or how to improve the event. One participant stated events “should be done the same way at all the churches” (57 y/o Marshallese female).

Most informal interview participants also described the trusted location of the event in the community as a facilitator in the overall decision to attend the vaccination event.

#### Subtheme: Influence of faith-based organization members and leaders

Marshallese participants more than Hispanic participants noted the influence of FBO leaders and members on their decision to get vaccinated. One Marshallese participant said, “I was scared…when I made my decision and got advice from our church leaders, the church members and I chose that it is the right thing for me to go get the COVID-19 vaccine” (57 y/o Marshallese female). Another Marshallese participant mentioned hearing from friends, “Our church was going to open for free vaccination” and “We need… to look for a healthy life for our family” (35 y/o Marshallese male). Fewer Hispanic participants in the formal interviews mentioned FBO factors as important in their decision-making, and only one mentioned the influence of FBO members and not FBO leaders. Marshallese and Hispanic informal interview participants frequently mentioned they heard about the event through their FBO. Numerous Hispanic informal interview participants mentioned that one Hispanic priest in particular influenced people to attend the event and get vaccinated.

### Institutional

#### Subtheme: Trust in healthcare organization and bilingual staff

Institutional factors, namely trust in the healthcare organization hosting the events and the bilingual staff, played a role in facilitating participants’ decisions to get vaccinated. Participants expressed trust in the healthcare organization hosting the community-engaged events. For example, one participant stated, “As a family-oriented community, we like to do it where we know the people in that place, and we can get right information about the vaccines or other questions we have” (54 y/o Marshallese female). When prompted about where they would receive booster vaccinations, participants responded, “The same department or the same organization that came to conduct the vaccine event. It’s because they came to help us with other services, and they did really welcome us to the vaccine event” (54 y/o Marshallese female).

Many participants discussed the importance of bilingual event staff. One participant who had his first dose at work stated, “The first time I took my first dose at [work]… I saw that some people were scared to come forward, they would just stand there not knowing who speaks Marshallese or who speaks English” (22 y/o Marshallese female). Another participant stated, “That was one of the most important things for me. Trust and above all, the same language, so that I can understand. And they can understand me, too” (28 y/o Hispanic female). Another participant had the same sentiment, noting, “We speak the same language” (54 y/o Hispanic female). Informal interview participants mentioned several institutional factors that facilitated vaccination, including the convenience of the location of the event, not needing an appointment, and the event being held at a convenient time (e.g., commonly evening or on the weekends). Informal interview notes indicated only one participant mentioned bilingual staff as a factor influencing their decision to attend the vaccination event.

### Policy

Policy-related factors were not mentioned by most formal interview participants. Only two participants mentioned workplace incentives and possible future vaccination mandates. One participant stated, “It just seems that it’s going to be mandated eventually along with other vaccines, so…might as well” (30 y/o Hispanic female). Another participant mentioned that people who get vaccinated at their workplace can “receive $200,” and therefore, “others are looking for their immunization cards or want to get vaccinated or are looking for places that still administered the vaccine” (35 y/o Marshallese male). The same participant mentioned, “My workplace was really happy when I didn’t take a day off” (35 y/o Marshallese male). Policy-related factors were more prominent in informal interview participants’ decisions to get vaccinated, specifically the lack of cost for vaccination as a facilitator.

## Discussion

This study’s Marshallese and Hispanic participants received a COVID-19 vaccine in late summer of 2021 when the vaccine had been available for more than a year, the Delta variant was prevalent, and ~ 67% of US adults had received at least one COVID-19 vaccination [24]. These late adopters chose to get vaccinated at community FBO vaccination events. SEM factors at the interpersonal, community, and institutional levels positively influenced participant and family attendance at events and decisions to get vaccinated.

### Intrapersonal

Late-adopting Marshallese and Hispanic participants mentioned concerns surrounding vaccine-related side effects, myths, and misconceptions about the COVID-19 vaccine. Fears that the vaccine had a chip in it were mentioned, although infrequently, and attributed to what ‘others’ say about the vaccine. Both infertility and concerns about a chip being implanted were reported in another qualitative study with Hispanic families [25], although the study did not report if participants were vaccinated. Scott and colleagues (2022) found that fear of chip insertion was a barrier to vaccination among Hispanic females; yet approximately half of the study participants reported intent to vaccinate against COVID-19 [26]. Concerns about the safety and myths and misconceptions of the COVID-19 vaccine were the top two reasons that unvaccinated participants were hesitant in a national survey with 225 Hispanic adults [27]. These results are also consistent with other studies [28] and scoping reviews of COVID-19 vaccine acceptance across different countries [15]. Participants in our study still chose to get vaccinated at FBOs despite concerns. Based on our analysis, other factors helped people overcome myths and misconceptions and get vaccinated.

### Interpersonal

Cultural values may have contributed to both Marshallese and Hispanic participants’ decisions to get vaccinated. Both cultures emphasize the needs of the family and/or community over the individual [9], [29]. Families often encompass multiple extended family members, and communal language such as “we” is used to speak in terms of family or community instead of the individual. Moore and colleagues’ (2022) study with a diverse sample of hesitant adopters, including Hispanic and Marshallese adults, found that the desire to protect family, friends, and the community was a driving force in vaccine decision-making [22]. Another qualitative study with Hispanic families found that the desire to protect family resulted in positive attitudes towards the vaccine [25], and the authors suggested that culturally focused vaccine campaigns should consider the entire Hispanic family unit. The desire to protect others is influential in COVID-19 vaccination uptake in other countries as well. A study in seven Arab countries [28] and a scoping review involving 19 countries [16] found the desire to protect oneself and relatives facilitates COVID-19 vaccine uptake. Conversely, Yuan & Chu (2022) conducted a study with a sample of ~ 70% of White individuals; respondents were more likely to have favorable attitudes towards the COVID-19 vaccine and mandates after viewing an individually-centered message compared with a community-centered message [30].

Participants frequently shared that they or their family members had a COVID-19 infection or that a family member had passed away from COVID-19, which prompted family decisions to get vaccinated. There are mixed results in the literature on the influence of exposure to death and/or prior illness from COVID-19 on vaccine hesitancy and uptake. A scoping review found that knowing someone who was infected with COVID-19 served as a facilitator to vaccine acceptance [16]. A survey of a diverse sample of 1,475 adults in Arkansas found that previous diagnosis with COVID-19 was not associated with COVID-19 vaccine hesitancy [31]. Another large US study found people who had family or friends with a prior infection or death from COVID-19 were less likely to refuse the COVID-19 vaccine [32]. In addition, a study of 615 adults in Naples, Italy found that people who had family or friends with past COVID-19 infections were less hesitant to receive a booster [33]. In contrast, two separate studies, one with African American participants [34] and another with both African American and Hispanic participants in the US South [35], found that the death of family or friends due to COVID-19 was a barrier rather than a facilitator to vaccination. Our study contributes to the literature as the first qualitative study among Marshallese and Hispanic participants suggesting that the death of a family member or having a prior COVID-19 infection positively influences the decision to be vaccinated. Based on our findings and the literature, it would be beneficial to investigate the impact of culturally tailored family-focused vaccine campaigns for different racial and ethnic communities. Motivational interviewing is a communication strategy that aims to support decision-making by aligning information with individuals’ values and beliefs and has been shown to decrease vaccine hesitancy [36].

### Community

At the community level, both Marshallese and Hispanic participants shared that FBOs were trusted locations which improved their comfort to attend the vaccination event. Our findings are consistent with literature demonstrating success in FBO-based health programs among Marshallese and Hispanic communities [29], [37], [38]. Conversely, informal interview participants frequently mentioned convenience as a reason for attending the event. Similarly, a survey in Arkansas found that the location of preference for COVID-testing among Hispanic adults is a community-based location in their neighborhood [39]. Marquez et al. (2021) also found that providing the COVID-19 vaccine at a central commercial and transport hub in the community was successful in vaccinating Hispanic community members, who most frequently reported getting vaccinated due to convenience in location and scheduling [40].

Marshallese participants shared that FBO leaders and members facilitated their decisions to get vaccinated. Our study confirms findings from previous research on vaccine hesitancy in Marshallese communities that highlight the importance of cultural and community leaders, particularly religious leaders, in encouraging vaccine uptake [41]. In contrast, Hispanic participants in our study did not report a strong influence from FBO leaders and members. The literature is mixed with regard to the influences of faith leaders among Hispanic communities regarding the COVID-19 vaccine. One study found that Hispanic adults in Arizona preferred COVID-19-related information from local leaders, faith-based leaders, and other community members [42]. Another study found that Hispanic individuals in the US Pacific Northwest preferred community leaders, advocacy groups, and community health workers to disseminate information about COVID-19 vaccines [25]. Marquez et al. (2021) relied on community leaders for targeted outreach and education in vaccination efforts with Hispanic participants. Approximately 20% of participants stated the most important decision-making factor in their attending the event was recommendation from a trusted source [40]. UAMS’ CBPR collaborative with Marshallese and Hispanic community members and leaders, through which trust developed over time, created the opportunity to rapidly respond to community needs during a pandemic. These results suggest that vaccine campaigns in Marshallese and Hispanic communities may benefit from strategies engaging trusted community and FBO members and leaders. Utilizing vaccinated community members as vaccine ambassadors to recruit friends and family members to get vaccinated has been shown to increase vaccine uptake [40].

### Institutional

At the institutional level, both Marshallese and Hispanic participants voiced trust in the healthcare organization and bilingual staff at the community-engaged events as facilitators in their decision-making. UAMS has a long-established partnership among the Marshallese and Hispanic communities [9], [29]. Our findings are consistent with Purvis and colleagues (2021), who found that local medical and academic institutions were sources of trust for vaccine information among a diverse sample of adults [41]. Similarly, interviews of 100 individuals in seven Arab countries and a scoping review which included studies from 19 different countries found that trust in the healthcare system was a facilitator to vaccination uptake [28]. The scoping review also noted that mistrust in the healthcare system served as a barrier to COVID-19 vaccine uptake, which, combined with our results and prior studies, emphasizes the importance of community and healthcare partnerships to support COVID-19 vaccination. Several studies have demonstrated that leveraging community partnerships in COVID-19 vaccination interventions and community outreach in native language result in increased vaccine uptake among minorities [25], [40], [13]. The importance of bilingual staff is also consistent with studies demonstrating that information which is not in a community’s native language is a barrier to COVID-19 vaccination and management [3], [43]. These studies highlight the collective importance of trusted organizations, community partnerships, and bilingual staff to support vaccination efforts among Marshallese and Hispanic communities.

### Policy

Policy factors were infrequently mentioned regarding Marshallese and Hispanic participants’ attendance at events or decisions to get the COVID-19 vaccine. Only one formal and one informal interview participant stated that a reason for getting the vaccine was that it was free. Few participants mentioned the possibility of vaccine mandates. The lack of policy level factors is in contrast to Moore and colleagues (2022), who found that employer-based vaccine mandates served as a motivator to hesitant adopters [22]. Similarly, vaccine policy served as a facilitator to vaccine uptake among 100 individuals in seven Arab countries [28]. Lack of policy factors as an impactful theme in our study may be due to the vaccines being provided free to participants without requiring appointments and lack of widespread vaccine mandates at the time of our study.

Although policies were not a facilitator or barrier to vaccine behaviors in our study, COVID-19 vaccine mandates have been implemented since our study was completed. As such, the multi-level complexities of supporting vaccination uptake among racial and ethnic minorities requires appropriate healthcare resources and policies, which is supported by the Immunization Agenda of 2030 [44]. Authors of a recent systematic review on factors affecting vaccination uptake among older ethnic minorities across numerous countries recommend that culturally tailored, multi-level approaches combining education, access, and interactions with trusted healthcare provides are needed to support vaccine policies and uptake among these underserved populations [45].

### Strengths and limitations

There are several strengths and limitations of this study. A limitation is the small sample size (9) for formal interviews. Additionally, participants who completed the formal interview were recruited from the large sample who completed the informal interview. However, the larger sample size of informal interviews was a strength and triangulated our findings. Another limitation is that our study sample was only from Arkansas, which may limit generalizability to Marshallese and Hispanic communities in other areas of the US. Also notable is that the increase in COVID-19 rates, hospitalizations, and deaths due to the Delta variant during the time this study took place likely played a factor in people’s decision to get vaccinated [46]. A strength of this study is our novel contribution to the literature about the socio-ecological influences among late-adopting Marshallese and Hispanic communities in the context of community-engaged vaccine events.

## Conclusion

Marshallese and Hispanic communities in the US have been disproportionately affected by COVID-19. Increasing vaccine uptake, especially among late adopters, is essential to address disparities. Based on our results and the literature, the following strategies may support vaccine uptake among Marshallese and Hispanic communities: (1) *interpersonal-level* – develop culturally-focused vaccine campaigns targeting the family units, (2) c*ommunity-level* – host vaccination events at convenient and/or trusted locations, such as FBOs, and engage community and/or FBO formal or lay leaders as vaccine ambassadors or champions, and (3) *institutional-level* – foster trust and a long-term relationship with the healthcare organization and provide bilingual staff at vaccination events. These results can inform future vaccine outreach interventions to improve health among Marshallese and Hispanic communities.

## Electronic Supplementary Material

Below is the link to the electronic supplementary material

Supplementary Material 1

Supplementary Material 2

## Data Availability

The deidentified data underlying the results presented in this study may be made available upon request from the corresponding author. The data are not publicly available in accordance with funding requirements and participant privacy.

## Abbreviations

CDC: Centers for Disease Control and Prevention
CBPR: community-based participatory research
UAMS: University of Arkansas for Medical Sciences
SEM: socio-ecological model
FBO: faith-based organizations
US: United States

## References

[1] Kriss JL, Hung MC, Srivastav A, et al. COVID-19 Vaccination Coverage, by Race and Ethnicity—National Immunization Survey Adult COVID Module, United States, December 2020–November 2021. *MMRW Morb Mortal Wkly Rep. *2022;71(23):757-763. doi:10.15585/mmwr.mm7123a2. 10.15585/mmwr.mm7123a2 PMC9181054 35679179

[2] Hooper MW, Nápoles AM, Pérez-Stable EJ. COVID-19 and Racial/Ethnic Disparities. *JAMA. *2020;323(24):2466–2467. doi:10.1001/jama.2020.8598. 10.1001/jama.2020.8598 PMC9310097 32391864

[3] Center KE, Da Silva J, Hernandez AL et al. Multidisciplinary Community-Based Investigation of a COVID-19 Outbreak among Marshallese and Hispanic/Latino Communities — Benton and Washington Counties, Arkansas, March–June 2020. *MMWR Morb Mortal Wkly Rep.* 2020;69(48):1807–1811. doi:10.15585/mmwr.mm6948a2. 10.15585/mmwr.mm6948a2 PMC7714036 33270609

[4] McElfish PA, Rowland B, Porter A, et al. Use of Community-Based Participatory Research Partnerships to Reduce COVID-19 Disparities among Marshallese Pacific Islander and Latino Communities–Benton and Washington Counties, Arkansas, April–December 2020. *Prev Chronic Dis. *2021;18:E91. doi:10.5888/pcd18.210124. 10.5888/pcd18.210124 PMC8522500 34618667

[5] Willis DE, McElfish PA. Racial Disparities in the COVID-19 Response Affecting the Marshall Islands Diaspora, United States of America. *Bull World Health Organ. *2021;99(9):680–681. doi:10.2471/BLT.20.277855. 10.2471/BLT.20.277855 PMC8381097 34475605

[6] Siegel M, Critchfield-Jain I, Boykin M, et al. Racial/Ethnic Disparities in State-Level COVID-19 Vaccination Rates and Their Association with Structural Racism. *J Racial Ethn Health Disparities*. 2022;9(6):2361-2374. doi:10.1007/s40615-021-01173-7 10.1007/s40615-021-01173-7 PMC8553106 34713336

[7] Ochieng C, Anand S, Mutwiri G, et al. Factors Associated with COVID-19 Vaccine Hesitancy among Visible Minority Groups from a Global Context: A Scoping Review. *Vaccines (Basel)*. 2021;9(12):1445. Published 2021 Dec 7. doi:10.3390/vaccines9121445 10.3390/vaccines9121445 PMC8708108 34960192

[8] Press VG, Huisingh-Scheetz M, Arora VM. Inequities in Technology Contribute to Disparities in COVID-19 Vaccine Distribution. *JAMA Health Forum*. 2021;2(3):e210264. doi:10.1001/jamahealthforum.2021.0264 10.1001/jamahealthforum.2021.0264 36218461

[9] McElfish PA, Kohler P, Smith C, et al. Community-Driven Research Agenda to Reduce Health Disparities. *Clin Transl Sci*. 2015;8(6):690-695. doi:10.1111/cts.12350 26573096 10.1111/cts.12350 PMC4703475

[10] McElfish PA, Cleek AB, Willis DE, et al. Leveraging Community Engagement Capacity to Address COVID-19 Disparities among Pacific Islander and Latinx Communities in Arkansas. *J Clin Transl Sci.* 2020;5(1):e81. Published 2020 Dec 7. doi:10.1017/cts.2020.562 10.1017/cts.2020.562 PMC8082130 34192048

[11] World Health Organization. Understanding the Behavioural and Social Drivers of Vaccine Uptake WHO Position Paper–May 2022. *Wkly Epidemiol Record*. 2022;97(20):209–24.

[12] Alagarsamy S, Mehrolia S, Pushparaj U, et al. Explaining the Intention to Uptake COVID-19 Vaccination Using the Behavioral and Social Drivers of Vaccination (BeSD) Model. *Vaccine X*. 2022;10:100140. doi:10.1016/j.jvacx.2021.100140 35013727 10.1016/j.jvacx.2021.100140 PMC8730788

[13] Osakwe ZT, Osborne JC, Osakwe N, et al. Facilitators of COVID-19 Vaccine Acceptance among Black and Hispanic Individuals in New York: A Qualitative Study. *Am J Infect Control*. 2022;50(3):268-272. doi:10.1016/j.ajic.2021.11.004 34793893 10.1016/j.ajic.2021.11.004 PMC8668153

[14] McLeroy KR, Bibeau D, Steckler A, et al. An Ecological Perspective on Health Promotion Programs. *Health Educ Q*. 1988;15(4):351-377. doi:10.1177/109019818801500401 3068205 10.1177/109019818801500401

[15] Al-Jayyousi GF, Sherbash MAM, Ali LAM, et al. Factors Influencing Public Attitudes towards COVID-19 Vaccination: A Scoping Review Informed by the Socio-Ecological Model. *Vaccines (Basel)*. 2021;9(6):548. doi:10.3390/vaccines9060548 10.3390/vaccines9060548 PMC8225013 34073757

[16] Lun P, Gao J, Tang B, et al. A Social Ecological Approach to Identify the Barriers and Facilitators to COVID-19 Vaccination Acceptance: A Scoping Review. *PLoS One*. 2022;17(10):e0272642. doi:10.1371/journal.pone.0272642 10.1371/journal.pone.0272642 PMC9529136 36191018

[17] Hallgren EA, McElfish PA, Rubon-Chutaro J. Barriers and Opportunities: A Community-Based Participatory Research Study of Health Beliefs Related to Diabetes in a US Marshallese Community. *Diabetes Educ*. 2015;41(1):86-94. doi:10.1177/0145721714559131 25398722 10.1177/0145721714559131 PMC4406271

[18] Creswell JW, Poth CN. *Qualitative Inquiry and Research Design: Choosing among Five Approaches*. 4th ed. Thousand Oaks, CA: SAGE Publications, Inc; 2016.

[19] Ritchie J, Lewis J, C McNaughton Nicolls, et al. *Qualitative Research Practice: A Guide for Social Science Students and Researchers*. 2nd ed. London: SAGE Publications Ltd; 2013.

[20] Kuzel A. Sampling in Qualitative Inquiry. In: Crabtree B, Miller W, editors. *Doing Qualitative Research*. Thousand Oaks, CA: SAGE Publications, Inc; 1992. p. 31–44.

[21] Centers for Disease Control and Prevention. CDC - BRFSS. Available: https://www.cdc.gov/brfss/index.html. Accessed 11 October 2022.

[22] Moore R, Purvis RS, Hallgren E, et al. Motivations to Vaccinate Among Hesitant Adopters of the COVID-19 Vaccine. *J Community Health*. 2022;47(2):237-245. doi:10.1007/s10900-021-01037-5 34687388 10.1007/s10900-021-01037-5 PMC8536476

[23] Gale NK, Heath G, Cameron E, et al. Using the Framework Method for the Analysis of Qualitative Data in Multi-Disciplinary Health Research. *BMC Med Res Methodol*. 2013;13:117. doi:10.1186/1471-2288-13-117 24047204 10.1186/1471-2288-13-117 PMC3848812

[24] KFF COVID-19 Vaccine Monitor. Kaiser Family Foundation. 2021. https://www.kff.org/coronavirus-covid-19/dashboard/kff-covid-19-vaccine-monitor-dashboard. Accessed July 2021.

[25] Garcia J, Vargas N, de la Torre C, et al. Engaging Latino Families About COVID-19 Vaccines: A Qualitative Study Conducted in Oregon, USA. *Health Educ Behav*. 2021;48(6):747-757. doi:10.1177/10901981211045937 34596462 10.1177/10901981211045937 PMC8581716

[26] Scott VP, Hiller-Venegas S, Edra K, et al. Factors Associated with COVID-19 Vaccine Intent among Latino SNAP Participants in Southern California. *BMC Public Health*. 2022;22(1):653. doi:10.1186/s12889-022-13027-w 35382803 10.1186/s12889-022-13027-w PMC8981200

[27] Piper BJ, Sanchez BV, Madera JD. Profiles of US Hispanics Unvaccinated for COVID-19. J Racial Ethn Health Disparities. 2022;10:553-559. doi:10.1007/s40615-022-01245-2 10.1007/s40615-022-01245-2 PMC8809210 35107819

[28] Elbarazi I, Yacoub M, Reyad OA, et al. Exploring Enablers and Barriers toward COVID-19 Vaccine Acceptance among Arabs: A Qualitative Study. *Int J Disaster Risk Reduct*. 2022;82:103304. doi:10.1016/j.ijdrr.2022.103304 36193257 10.1016/j.ijdrr.2022.103304 PMC9519527

[29] McElfish PA, Moore R, Laelan M, et al. Using CBPR to Address Health Disparities with the Marshallese Community in Arkansas. *Ann Hum Biol*. 2018;45(3):264-271. doi:10.1080/03014460.2018.1461927 29877159 10.1080/03014460.2018.1461927

[30] Yuan S, Chu H. Vaccine for Yourself, Your Community, or Your Country? Examining Audiences’ Response to Distance Framing of COVID-19 Vaccine Messages. *Patient Educ Couns.* 2022;105(2):284-289. doi:10.1016/j.pec.2021.08.019 34479746 10.1016/j.pec.2021.08.019 PMC8384529

[31] Willis DE, Selig JP, Andersen JA, et al. Hesitant but Vaccinated: Assessing COVID-19 Vaccine Hesitancy among the Recently Vaccinated. *J Behav Med.* 2022;1-10. doi:10.1007/s10865-021-00270-6 10.1007/s10865-021-00270-6 PMC8760868 35032254

[32] Khubchandani J, Sharma S, Price JH, et al. COVID-19 Morbidity and Mortality in Social Networks: Does It Influence Vaccine Hesitancy? *Int J Environ Res Public Health*. 2021;18(18):9448. doi:10.3390/ijerph18189448 10.3390/ijerph18189448 PMC8470484 34574373

[33] Folcarelli L, Miraglia Del Giudice G, Corea F, et al. Intention to Receive the COVID-19 Vaccine Booster Dose in a University Community in Italy.* Vaccines (Basel)*. 2022;10(2):146. doi:10.3390/vaccines10020146 10.3390/vaccines10020146 PMC8877002 35214605

[34] Willis DE, Andersen JA, Montgomery BEE, et al. COVID-19 Vaccine Hesitancy and Experiences of Discrimination among Black Adults. *J Racial Ethn Health Disparities*. 2022;1-10. doi:10.1007/s40615-022-01290-x 10.1007/s40615-022-01290-x PMC8989097 35391714

[35] Bateman LB, Hall AG, Anderson WA, et al. Exploring COVID-19 Vaccine Hesitancy among Stakeholders in African American and Latinx Communities in the Deep South through the Lens of the Health Belief Model. *Am J Health Promot*. 2022;36(2):288-295. doi:10.1177/08901171211045038 10.1177/08901171211045038 PMC8770578 34719985

[36] Coley KC, Gessler C, McGivney M, et al. Increasing Adult Vaccinations at a Regional Supermarket Chain Pharmacy: A Multi-Site Demonstration Project. *Vaccine*. 2020;38(24):4044-4049. doi:10.1016/j.vaccine.2020.02.040 32093985 10.1016/j.vaccine.2020.02.040

[37] Derose KP, Rodriguez C. A Systematic Review of Church-Based Health Interventions among Latinos. *J Immigr Minor Health*. 2020;22(4):795-815. doi:10.1007/s10903-019-00941-2 31654217 10.1007/s10903-019-00941-2 PMC7182479

[38] McElfish P, Bing W, Ayers BL, et al. Lessons Learned through a Partnership with Marshallese Faith-Based Organizations to Screen for Hypertension and Diabetes. *J Reg Med Campuses*. 2018;1(3). doi:10.24926/jrmc.v1i3.1044

[39] McElfish PA, Willis DE, Bryant-Moore K, et al. Arkansans’ Preferred COVID-19 Testing Locations. *J Prim Care Community Health*. 2021;12:21501327211004289. doi:10.1177/21501327211004289 33771056 10.1177/21501327211004289 PMC8767652

[40] Marquez C, Kerkhoff AD, Naso J, et al. A Multi-Component, Community-Based Strategy to Facilitate COVID-19 Vaccine Uptake among Latinx Populations: From Theory to Practice. *PLoS One*. 2021;16(9):e0257111. doi:10.1371/journal.pone.0257111 10.1371/journal.pone.0257111 PMC8452046 34543291

[41] Purvis RS, Hallgren E, Moore RA, et al. Trusted Sources of COVID-19 Vaccine Information among Hesitant Adopters in the United States. *Vaccines (Basel)*. 2021;9(12):1418. doi:10.3390/vaccines9121418 10.3390/vaccines9121418 PMC8706404 34960164

[42] Ignacio M, Oesterle S, Mercado M, et al. Narratives from African American/Black, American Indian/Alaska Native, and Hispanic/Latinx Community Members in Arizona to Enhance COVID-19 Vaccine and Vaccination Uptake. *J Behav Med*. 2022;1-13. doi:10.1007/s10865-022-00300-x 10.1007/s10865-022-00300-x PMC8942760 35322313

[43] Deal A, Hayward SE, Huda M, et al. Strategies and Action Points to Ensure Equitable Uptake of COVID-19 Vaccinations: A National Qualitative Interview Study to Explore the Views of Undocumented Migrants, Asylum Seekers, and Refugees. *J Migr Health*. 2021;4:100050. doi:10.1016/j.jmh.2021.100050 34075367 10.1016/j.jmh.2021.100050 PMC8154190

[44] Olayinka F, Sauer M, Menning L, et al. Building and Sustaining Public and Political Commitment to the Value of Vaccination: Recommendations for the Immunization Agenda 2030 (Strategic Priority Area 2). *Vaccine*. 2022;S0264-410X(22)01451-7. doi:10.1016/j.vaccine.2022.11.038 10.1016/j.vaccine.2022.11.038 PMC11413528 36528448

[45] Bhanu C, Gopal DP, Walters K, et al. Vaccination Uptake amongst Older Adults from Minority Ethnic Backgrounds: A Systematic Review. *PLoS Med*. 2021;18(11):e1003826. doi:10.1371/journal.pmed.1003826 34735440 10.1371/journal.pmed.1003826 PMC8568150

[46] Masters NB, Zhou T, Meng L, et al. Geographic Heterogeneity in Behavioral and Social Drivers of COVID-19 Vaccination. *Am J Prev Med*. 2022;63(6):883-893. doi:10.1016/j.amepre.2022.06.016 36404022 10.1016/j.amepre.2022.06.016 PMC9296705

