# Supplementary material for: Reaching late adopters: factors influencing COVID-19 vaccination of Marshallese and Hispanic adults
Source: BMC Public Health. 2023 Apr 3;23:631. doi: 10.1186/s12889-023-15468-3 (PMC10068695; doi:10.1186/s12889-023-15468-3)
Supplement: Supplementary file 1 — Supplementary Material 1 [file 12889_2023_15468_MOESM1_ESM.docx]

**Additional File Information**

**File name:** Additional File 1

**File format:** Word

**Title of data:** Informal Interview Guide for Vaccine Event Participants

**Description of data:** The informal interviews with event participants were brief (3-5 minutes average duration), and included the following questions: 1) reasons for attending, 2) COVID-19 vaccine decision-making, 3) barriers/facilitators to attendance, 4) recommendations for event improvement, and 5) general thoughts about COVID-19 vaccines.

**Additional File 1**

**Informal Interview Guide for Vaccine Event Participants**

These interviews are intended to be very brief and conversational in nature (1-5 minutes). There is no formal interview guide. The questions below are *suggestions*. Use whatever wording you choose. We are trying to gain a general understanding of the role these events are playing in the effort to vaccinate community members. We don’t need to speak with everyone who attends these events. Try to get a mixture of the kinds of people attending. Be respectful of people’s time and space. You do not need to be “in private” to have these conversations, but be discrete and try not to have the conversations overheard. Here are some steps to follow:

**Step 1: Gain permission to ask a few questions**

- “I am here to with UAMS, one of the event sponsors. Can I ask you a few questions about your coming here today? It will only take a few minutes.”

**Step 2: Ask questions**

- These are the topics we are interested in:
  - Why they came to *this* event
    - What was attractive about this event?
    - How did they hear about it?
    - Had they considered other ways of getting vaccinated? Which?
  - How they decided to be vaccinated
    - Discussions with family, friends, health care providers?
    - Other sources of information they accessed?
  - Barriers/facilitators to attending this event
    - Feasibility of getting there, when it was scheduled, etc.
    - “What worked well/not about the event”?
  - What could make attending an event like this easier/better?
  - General thoughts about COVID-19 vaccines
    - Have they been hesitant? What have been their concerns? What “changed their mind”?
- Remember: Only talk as long as people seem comfortable. Don’t push. Any small exchange will be helpful.

**Step 3: Seek permission to contact later for a more formal interview**

- We only need permission from 3-5 participants per event.
- Try to get a contact name and phone # to schedule a formal interview later on (within a month ideally).
- “How would you feel about having a bit longer conversation on the phone about your experience? This has been so helpful, and we’d love to hear more from you. And we can provide a gift card for your efforts.”

**Step 4: Document each informal interview**

- Create a brief summary of the conversation. Bullet points. Interviewers will verbally debrief after each events.
