# Supplementary material for: Reaching late adopters: factors influencing COVID-19 vaccination of Marshallese and Hispanic adults
Source: BMC Public Health. 2023 Apr 3;23:631. doi: 10.1186/s12889-023-15468-3 (PMC10068695; doi:10.1186/s12889-023-15468-3)
Supplement: Supplementary file 2 — Supplementary Material 2 [file 12889_2023_15468_MOESM2_ESM.docx]

**Additional File Information**

**File name:** Additional File 2

**File format:** Word

**Title of data:** Semi-Structured Interview Guide

**Description of data:** The semi-structured interview guide explored participant’s thoughts on the COVID-19 vaccine, vaccine decision-making, reasons for choosing to attend and get vaccinated at the community location, and recommendations for event improvement.

**Additional File 2**

*Semi-Structured Interview Guide*

| - What are your overall thoughts about the COVID-19 vaccine(s)? - How effective do you think they are? - How do they compare to other vaccines that you have heard about? - Why did you select to get a COVID-19 vaccine? - How did you go about deciding to get a vaccine? - Talk with others? Guidance from someone specific (e.g., pastor)? Read about? - Did anyone recommend that you get the COVID-19 vaccine(s)? - If so, who? What did they say? - Did anyone discourage you from getting the COVID-19 vaccine(s)? - If so, who? What did they say? - Before getting vaccinated, what kinds of questions did you want answered about the COVID-19 vaccine(s)? - Why do you think that some people choose to not get a COVID-19 vaccine? - Why did you choose to get vaccinated at the community event?   - How did you hear about it?   - What about the event appealed to you? - Why did you choose the event over another option? (pharmacy/clinic…) - How would you describe your experience at the COVID-19 vaccine event? - What do you think worked well at the event?   - What do you think could have been done better at the event? - If it turns out that additional COVID-19 vaccines will be recommended in the future (like annual flu shots), what will you do?   - Be vaccinated again/not? Why? - If you go get another vaccine, where would you like to get it? Why? - Do you have any recommendations for adapting vaccine delivery or other medical intervention events to better fit the community’s needs? - Are there any specific actions that UAMS or community organizations could take to make the vaccine events like the one you went to more successful? - Is there anything about the COVID-19 vaccine or the vaccination event you went to that you would like to share or talk about that was not asked? |
| --- |
